# Identifying High-Risk Neighborhoods Using Electronic Medical Records: A Population-Based Approach for Targeting Diabetes Prevention and Treatment Interventions

**DOI:** 10.1371/journal.pone.0159227

**Published:** 2016-07-27

**Authors:** Rose Gabert, Blake Thomson, Emmanuela Gakidou, Gregory Roth

**Affiliations:** 1 University of Washington, Seattle, United States of America; 2 University of Cambridge, Cambridge, United Kingdom; Geisel School of Medicine at Dartmouth College, UNITED STATES

## Abstract

**Background:**

Increasing attention is being paid to the marked disparities in diabetes prevalence and health outcomes in the United States. There is a need to identify the small-area geographic variation in diabetes risk and related outcomes, a task that current health surveillance methods, which often rely on a self-reported diagnosis of diabetes, are not detailed enough to achieve. Broad adoption of electronic health records (EHR) and routine centralized reporting of patient-level data offers a new way to examine diabetes risk and highlight hotspots for intervention.

**Methods and Findings:**

We examined small-area geographic variation in hemoglobin A1c (HgbA1C) levels in three counties though a retrospective observational analysis of the complete population of diabetic patients receiving at least two ambulatory care visits for diabetes in three counties (two urban, one rural) in Minnesota in 2013, with clinical performance measures re-aggregated to patient home zip code area. Patient level performance measures included HgbA1c, blood pressure, low-density lipoprotein cholesterol and smoking. Diabetes care was provided to 63,053 patients out of a total population of 1.48 million people aged 18–74. Within each zip code area, on average 4.1% of the population received care for diabetes. There was significant and largely consistent geographic variation in the proportion of patients within their zip code area of residence attaining HgbA1C <8.0%, ranging from 59–90% of patients within each zip code area (interquartile range (IQR) 72.0%-78.1%). Attainment of performance measures for a zip code area were correlated with household income, educational attainment and insurance coverage for the same zip code area (all p < .001).

**Conclusions:**

We identified small geographic areas with the least effective control of diabetes. Centrally-aggregated EHR provides a new means of identifying and targeting at-risk neighborhoods for community-based interventions.

## Introduction

Increasingly, policy makers and public health officials are calling for patient-centered health interventions that are based in communities rather than clinics in order to prevent and treat cardiometabolic risk factors. [1, 2] Unfortunately, data on disease and risk factors is lacking for small geographic areas; sample-sizes of large health exam surveys do not allow for identifying the specific neighborhoods where at-risk individuals live and biomarkers like HgbA1C remain difficult to obtain as part of routine surveillance. [3,4] Without high-quality data on where people at highest risk from diabetes actually live, interventions are tested across broader areas with lower average risk, which in turn leads to negative results or minimal measured effect sizes for these trials. In turn, the evidence for high-quality interventions to prevent and treat diabetes remains extremely limited. [5]

Minnesota Community Measurement (MNCM) is a non-profit organization that receives patient-level data from electronic health records (EHRs) for almost all ambulatory clinics in Minnesota, as mandated by the Minnesota Legislature. [6] Using this data, we sought to better understand geographic variation in achieved levels of HgbA1c among diabetics at the level of zip code areas. This analysis served as the needs assessment to inform selection of interventions that will be implemented as part of the HealthRise program. HealthRise is a 5-year initiative to evaluate community-based interventions to improve cardiometabolic health in the United States, India, Brazil and South Africa. [7]

## Methods

### Data Source

Three sites in Minnesota were selected for the United States component of the HealthRise program: Hennepin County (Minneapolis), Ramsey County (St. Paul) and rural Rice County. These sites were selected as representative of urban and rural regions with populations in need of improved diabetes prevention in the United States.

Data was obtained from MNCM, which receives clinical data from electronic health record systems on all patients visiting eligible providers for diabetes care in Minnesota. For this analysis, MNCM aggregated data by residential zip code areas and made it available as age-sex-race stratified tables with counts of patients achieving selected performance measures. We included patients meeting the following criteria: a diagnosis of diabetes mellitus based on ICD9 codes 250.00–250.93; age 18 to 75 on January 1, 2013; and at least two outpatient visits with an eligible provider between January 1, 2012 and December 31, 2013. ICD-9 codes could be in any position in the record. Eligible providers include physicians holding MD or DO degrees, physician assistants, or nurse practitioners. Patients were excluded if they were pregnant, enrolled in hospice, living in a nursing home, or if they died during the measurement period. Data was not collected from prisons, federal hospitals or from facilities run by the Indian Health Service (IHS). To ensure patient anonymity, data were not reported for observations in any cell with fewer than 15 patients. The U.S. Census Bureau’s American Community Survey 5-year estimates from 2013 were used to provide population data by zip code area. [8]

### Analysis

We examined the four major clinical measures of cardiometabolic health among diabetic patients: reduction of blood glucose, reduction of blood pressure, reduction of low-density lipoprotein (LDL) cholesterol, and avoidance of tobacco smoking. Achievement of performance measures was defined by MNCM as HgbA1c <8.0%, LDL-cholesterol <100 mg/dL, systolic blood pressure <140 mmHg and diastolic blood pressure <90 mmHg. Achievement of the tobacco smoking indicator was defined as clinical documentation that a patient was currently a non-smoker. As the primary outcome of interest, we calculated the proportion of patients meeting performance measures within each zip code area by dividing the number of patients meeting the target by the total number of patients within that zip code area. To summarize zip code area performance as a single value for all measures, we assigned a rank to each zip code area based on performance on each individual indicator and calculated the mean rank across the four clinical indicators. The resulting rank value serves as a measure of relative performance at the level of a zip code area. We computed the Spearman Rank correlation between proportion of patients meeting targets and sociodemographic variables defined at the zip code area level. All statistical analyses were performed using Stata 13.1 and all geographic analyses were performed using ArcMap version 9.3.1. [9, 10] The study received institutional review board (IRB) approval from the University of Washington.

## Results

Out of a total population of 1.48 million people aged 18–74 living in 120 zip code areas, 63,053 (4.3%) were identified in our data as having received regular primary care for diabetes in 2013. The median prevalence for a diabetic receiving care within each zip code area was 4.1%, (interquartile range (IQR) 3.1–4.8%) (Table 1). Slightly more than half of diabetic patients were male, 68.2% were identified as white race and two-thirds of diabetic patients were aged 55–75. Only three-quarters of patients achieved an HgbA1C <8.0% with slightly higher rates for achieving blood pressure targets. Current tobacco smoking was reported for 15% of diabetic patients. An LDL-cholesterol < 100 mg/dl was achieved by only a third.

**Table 1:** Descriptive Statistics of the Patient Population and Zip Code Areas of Residence.

Study population		
Patients (n)	63,053	
Zip code areas (n)	120	
Age 35–54 (n, %)	18,585	32.8
Age 55–75 (n, %)	38,108	67.2
Male (n, %)	33,011	52.5
Female (n, %)	29,861	47.5
White race (n, %)	41,758	68.2
Nonwhite race (n, %)	19,509	31.8
Patients per zip code area (median, IQR)	567	179, 894
Percent of population receiving diabetes care (median %, IQR)	4.1	3.1, 4.8
Achieving HbA1c <8.0%, n = 46,834 (median %, IQR)	75.6	72.0, 78.1
Achieving BP <140/90, n = 54,154 mmHg (median %, IQR)	86.7	83.9, 88.9
Achieving LDL-cholesterol <100 mg/dL, n = 41,976, (median %, IQR)	67.3	64.2, 70.6
Achieving No tobacco smoking, n = 53,943, (median %, IQR)	87.2	83.3, 90.0

IQR = interquartile range. IQR values represent variation across zip code areas.

**Fig 1:**
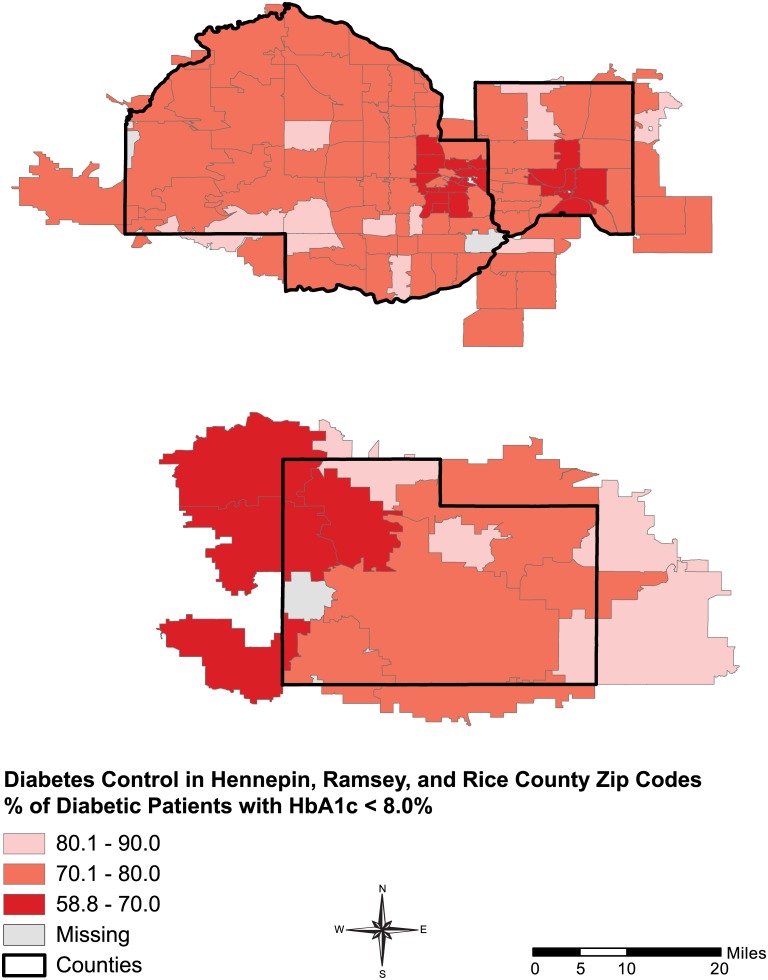
Percent of diabetic patients achieving HbA1c <8.0%.

There was significant geographic variation in the proportion of patients within a zip code area attaining a HgbA1C <8.0%, ranging from 59–90% across zip code areas (IQR 72.0%-78.1%) (Fig 1). Areas with the smallest proportion of patients meeting their HgbA1c target were clustered in the urban areas of Hennepin and Ramsey (The Minneapolis-St. Paul corridor) and more rural areas of Rice County. Similar patterns were seen for the other performance measures (Figs A–C in S1 Appendix).

**Fig 2:**
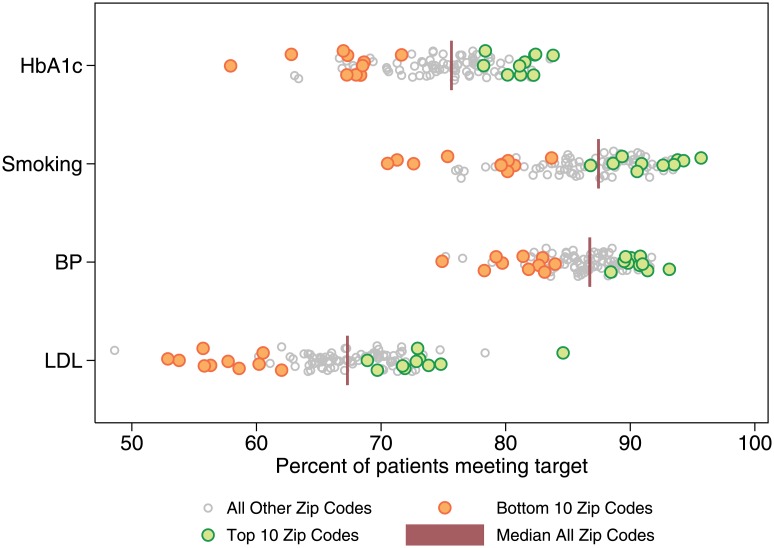
Distribution of Zip Code Area Performance with Top and Bottom 10 Zip Code Areas by Summary Score.

The same zip code areas tended to perform poorly across all four indicators, with 8 of the 10 zip code areas ranked lowest overall also in the lowest quintile for all 4 clinical quality indicators individually. Similarly, nine of the 10 highest-ranking zip code areas fell within the top quintile for at least 3 of the measures and above the mean for all of them. The distribution of zip code area-level performance was broadest for LDL-cholesterol and narrowest for blood pressure. The distribution of zip code area performance by measure is shown in Fig 2, with the highest- and lowest-ranking zip code areas identified according to their average rank across all measures.

Using census data, we examined the correlation between the percentage of diabetic residents achieving performance measures within a zip code area and measures of socioeconomic status (SES) (Fig B in S1 Appendix). The blood glucose performance measure was significantly positively correlated with all three zip code area-level indicators of SES (median household income, percent completing high school, and percent with insurance coverage). The Spearman rank correlation coefficient for income, education and insurance coverage were 0.66 [p < .001], 0.70 [p < .001], and 0.63 [p < .001], respectively. These SES indicators were even more strongly associated with the proportion of patients abstaining from smoking, with Spearman correlation coefficients of 0.79 [p < .001], 0.72 [p < .001], and 0.70 [p < .001], respectively. The correlation with blood pressure and LDL-cholesterol were weaker but still significant (0.42 [p < .001], 0.42 [p < .001], and 0.40 [p < .001] and 0.55 [p < .001], 0.45 [p < .001], and 0.39 [p < .001], respectively).

## Discussion

Using electronic health record data, we identified the small number of neighborhoods where targets for HgbA1c, blood pressure, LDL-cholesterol and tobacco cessation were least commonly achieved among diabetic patients. When examined by a patient’s zip code area of residence, these clinical performance measures were strongly correlated with the average level of income, education, and insurance coverage in that zip code area. Our approach offers a new way to identify the areas most at risk due to diabetes and can guide the design of studies in patient-centered community-based cardiometabolic risk factor modification.

Our analysis adds to the existing evidence showing disparities in the prevalence of risk factors, disease, and poor clinical outcomes by showing the effect of a patient’s home neighborhood on diabetes outcomes. In line with previous publications, we’ve demonstrated that disease clustering is found not only among sociodemographic groups but also neighborhood geographies. [11–14] As the pressure for community-based interventions intensifies, it becomes more important than ever that we are able to identify those communities at highest risk of disease and complications and design appropriate interventions based on their unique disease and risk factor profile.

Studies of regional variation in diabetes care have often relied on Medicare data or reports of Healthcare Effectiveness Data and Information Set (HEDIS) quality measures and focused on the influence of race and geography in general. Most studies have relied on aggregate data representing large geographic regions, such as hospital referral regions, counties, states or the entire country. [1–4, 11–14] Larger-scale population-wide survey studies capture overall trends, but are limited in their ability to narrow-in on smaller geographies: biomarkers in the NHANES survey are representative only at the multi-state or national level, the Behavioral Risk Factor Surveillance Survey is limited to self-report; in Minnesota itself, county health surveys are usually only self-report and consist of aggregated “health areas” smaller than counties but still too large for the identification of community-level interventions. [15–18] These data sources, though meant to inform public health action, obscure important disparities in health at the most local level. In contrast, our analysis includes almost the entire patient population and shows small-area variation in achievement of quality indicators according to where patients live, not where they seek care. Our results suggest an opportunity to apply more advanced geostatistics, such as Kriging and measurement of spatial autocorrelation, to enhance the usefulness of electronic health record data.

Based on these analyses, we were able to identify 7 heavily burdened zip code areas burdened by a disproportionately low level of control for cardiometabolic risk factors. This data was compiled and shared with local stakeholders including the Minnesota Department of Health, County and City Departments of Health, local accountable care organizations and other health care providers, non-governmental organizations, and other public health experts. As part of the HealthRise project, the analysis was used by local stakeholders to design community-based interventions that will be implemented and evaluated across high risk zip code areas over the next three years. MNCM data could also be used to evaluate the impact of these interventions.

Our analysis was limited by important patient anonymity safeguards: Minnesota Community Measurement provided tabulated data rather than patient-level microdata, and all cells with fewer than 15 people at any level of aggregation were excluded. To avoid bias, we restricted our analysis to zip code areas where the subpopulations of interest were non-missing for groups of interest (for instance, White and non-White). The observed correlations between HgbA1C and zip code area characteristics are ecological and hypothesis-generating in nature rather than proof of a causal relationship. However, the use of biomarker outcomes including HgbA1c, blood pressure level and LDL-cholesterol level rather than self-reported diagnosis or diagnosis billing codes should provide some reassurance regarding the measurement of prevalent risk factors and disease.

There is a growing need for community-based interventions that can prevent diabetes and address persistent disparities in health outcomes. To show even a moderate-sized effect, studies of these interventions will need to be tested in small geographic areas where blood glucose and other risk factors are highest and adverse health outcomes are common. We have shown that aggregated electronic health records offer a novel approach for identifying these small, high risk neighborhoods that are missed by the larger regions identified in health examination surveys. Our results suggest that regularly-collected EHR data may be a useful, low-cost approach for identifying the hotspots where diabetes prevention programs can have the largest impact.

## Supporting Information

S1 Appendix **Figs A, B, C**: Percentage of patient population achieving other performance measures by zip code area. **Figure D**: Correlation of HbA1c control with socioeconomic status variables.
(DOCX)
