# Supplementary material for: Identifying High-Risk Neighborhoods Using Electronic Medical Records: A Population-Based Approach for Targeting Diabetes Prevention and Treatment Interventions
Source: PLoS One. 2016 Jul 27;11(7):e0159227. doi: 10.1371/journal.pone.0159227 (PMC4963128; doi:10.1371/journal.pone.0159227)

Supporting Materials: Supplementary Appendix 1

Table of Contents:

Figures A, B, C Page 1

Percentage of patient population achieving performance measure by zip code

Figure D Page 5

Correlation of HbA1c control with socioeconomic status variables

Figure A: Percentage of Patient Population Achieving BP Target by Zip Code Areas
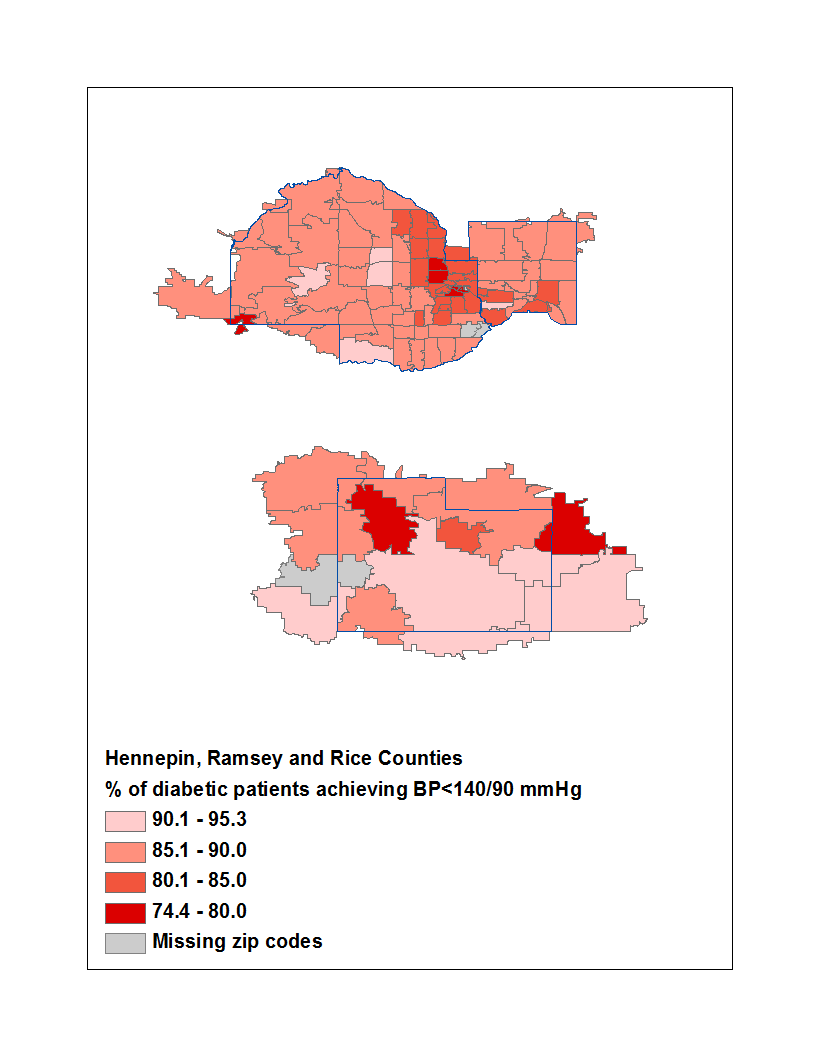


Figure B: Percentage of Patient Population Achieving LDL-C Target by Zip Code Areas
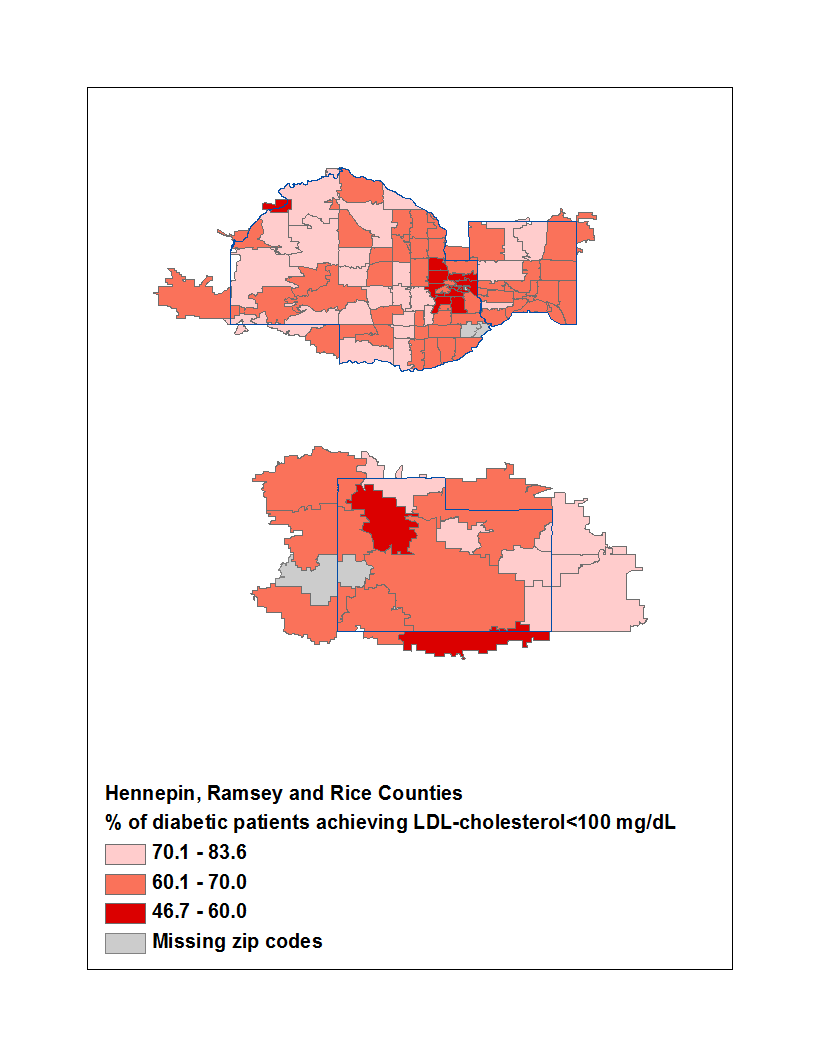


Figure C: Percentage of Patient Population Achieving Smoking Target by Zip Code Areas
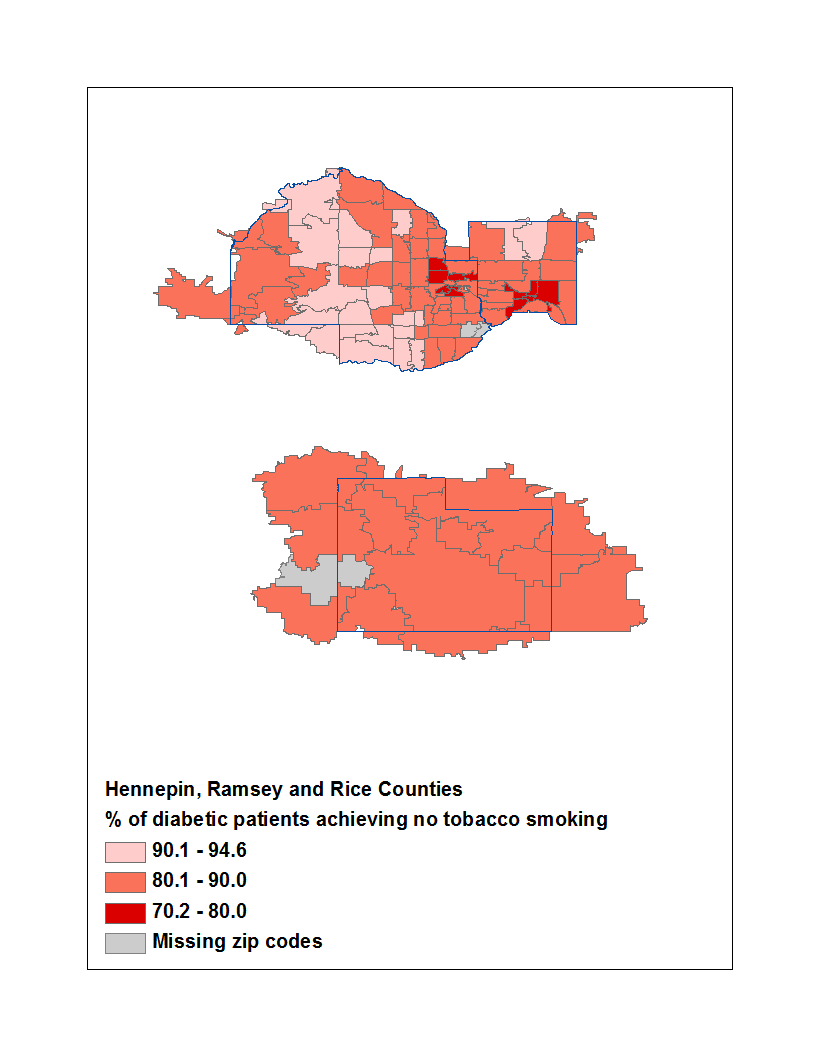


Figure D. Association of HbA1c control and socioeconomic variables within zip code areas


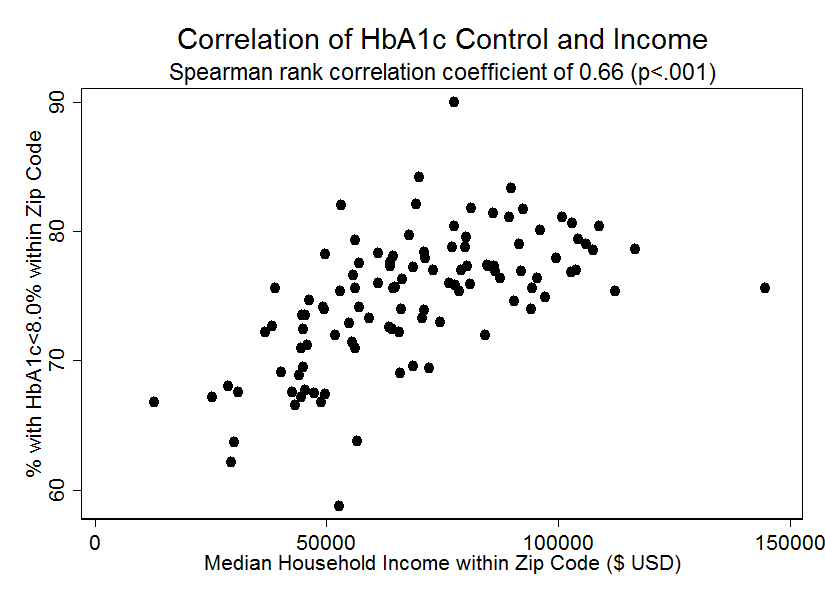

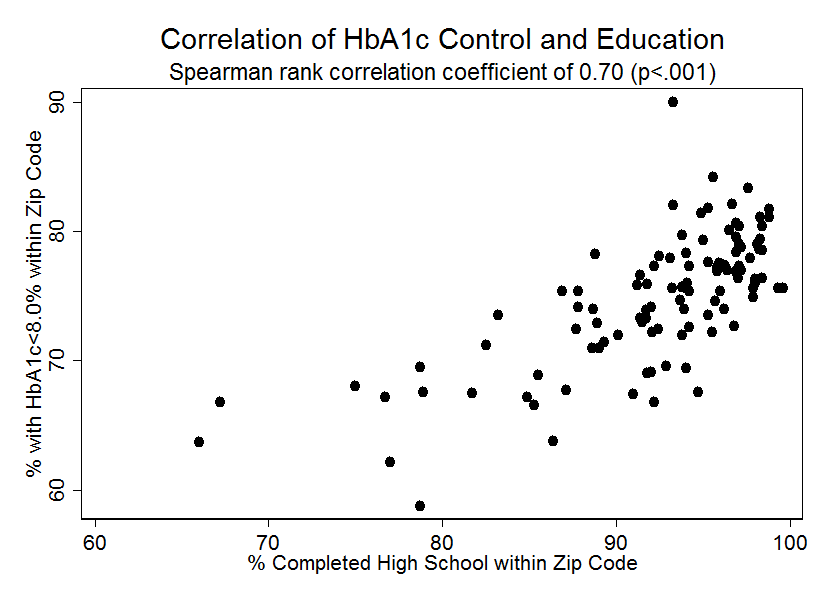


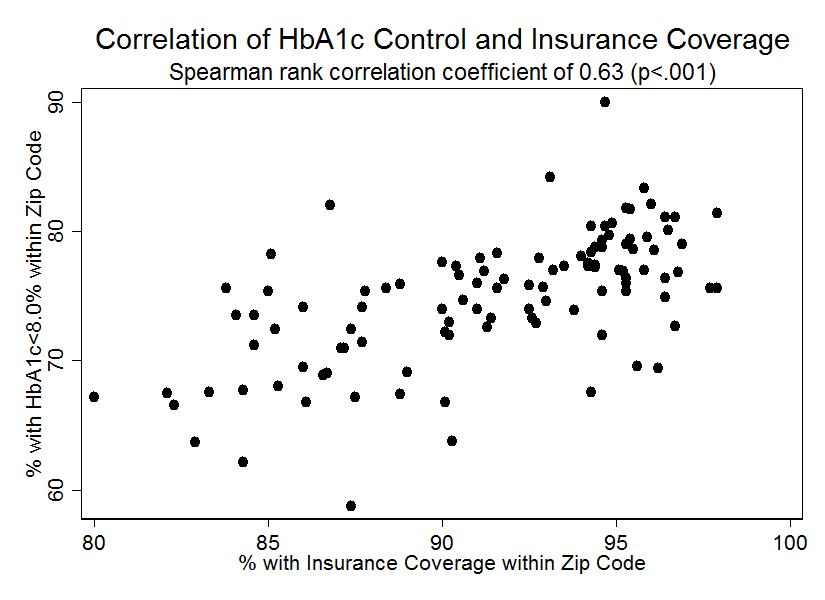

Supplement: S1 Appendix — Figs A, B, C: Percentage of patient population achieving other performance measures by zip code area. Figure D: Correlation of HbA1c control with socioeconomic status variables. (DOCX) [file pone.0159227.s001.docx]
